# Hypervolemic Hyponatremia as a Reversible Cause of Cardiopulmonary Arrest in a Postpartum Patient with Preeclampsia

**DOI:** 10.1155/2021/8850725

**Published:** 2021-02-04

**Authors:** Richard Hsu, Anna Tong, Chaur-Dong Hsu

**Affiliations:** ^1^Wayne State University School of Medicine, Detroit, Michigan, USA; ^2^Department of Obstetrics and Gynecology, Detroit Medical Center, Detroit, MI, USA; ^3^Department of Obstetrics and Gynecology, Wayne State University School of Medicine, Detroit, MI, USA; ^4^Department of Physiology, Wayne State University School of Medicine, Detroit, MI, USA

## Abstract

Although the incidence of preeclampsia complicated by hyponatremia is reportedly rare, the effects on the maternal outcome are severe and life-threatening. Here, we describe a case of a patient with preeclampsia who coded postpartum and was discovered to have hypervolemic hyponatremia and subsequently recovered after fluid diuresis and resolution of hyponatremia. While hyponatremia in preeclampsia is rare, it is even more unique for it to lead to cardiopulmonary arrest consequently. Therefore, sodium levels and fluid status should be monitored closely and promptly corrected without delay to prevent cardiopulmonary arrest in patients with preeclampsia.

## 1. Introduction

Preeclampsia is defined as a systolic blood pressure ≥ 140 mmHg or a diastolic blood pressure ≥ 90 mmHg measured on two separate occasions greater than 4 hours apart, with evidence of proteinuria of ≥300 mg, in a pregnant woman after 20 weeks of gestation [1]. Preeclampsia has hypertension and proteinuria and symptoms such as edema, visual disturbances, headache, and epigastric pain. The risk of developing hyponatremia as a complication of preeclampsia has been reported in several cases. Proteinuria and blood loss from delivery have an impact on osmotic status, leading to decreased perfusion and, subsequently, fluid overload. Here, we describe a case of a patient with preeclampsia who coded postpartum and was discovered to have hypervolemic hyponatremia and subsequently recovered after fluid diuresis and resolution of hyponatremia. This particular case highlights the importance of close monitoring and early detection of hyponatremia as a preventable cause of cardiopulmonary arrest in patients with preeclampsia.

## 2. Case Presentation

A 38-year old female, G10P4054, 30 weeks and 1 day, with a BMI of 32.4 kg/m^2^ (weight: 85 kg, height: 162 cm) with no prenatal care and a history of uncontrolled chronic hypertension and medication noncompliance, presented with severe abdominal pain without vaginal bleeding. On admission, the patient was noted to have elevated blood pressures (220/133, 210/130, and 191/116 mmHg), which were acutely managed with intravenous labetalol (20, 40, and 80 mg) and subsequently with hydralazine (10 mg, 10 mg). The patient otherwise was asymptomatic and reported regular fetal movements. Her pregnancy course was otherwise uncomplicated thus far.

Upon history intake, the patient was found to have not received any prenatal care and had uncontrolled chronic hypertension diagnosed at age 26. Furthermore, due to her labile blood pressures, elevated transaminases (ALT 56 U/L; AST 50 U/L; LDH 343 U/L), and thrombocytopenia (platelet count ranging from 101 to 113 K/mm^3^), the patient was diagnosed with chronic hypertension with superimposed preeclampsia. For management, the patient received the first dose of betamethasone for fetal lung maturation and magnesium sulfate for seizure prophylaxis and neuroprotection. Fetal heart rate tracings demonstrated a baseline of 130 bpm with moderate variability, accelerations present, and no decelerations. Maternal-fetal medicine was consulted and recommended that if the patient continued to have severe range blood pressures, or progressively worsening preeclamptic labs or symptomatology, then proceed with delivery. It was also recommended to monitor intake and output values carefully.

Within six hours, the patient's blood pressure increased to 160-180/90-100 mmHg. Labetalol was given, and the patient's blood pressure decreased to 140-150/60-80 mmHg. The patient's net intake and output were recorded as +516 mL on the first day of admission.

On hospital day 2, the patient received her second dose of betamethasone and was noted to have increased blood pressures again to 170-180/90-100 mmHg that late afternoon. Thus, a decision was made to proceed with an urgent primary low transverse cesarean delivery. After cesarean delivery of a 1195 g female infant, the patient had an estimated blood loss of 590 mL and a subsequent decrease in hemoglobin postoperatively from 11.8 to 9.7 mg/dL. The patient was subsequently given 30 U of continuous oxytocin in 1000 mL normal saline infusion at 125 mL/h postpartum as a uterotonic and postpartum magnesium sulfate for seizure prophylaxis. The patient's intake and output net was recorded as +5981 mL (see Table 1).

**Figure 1:**
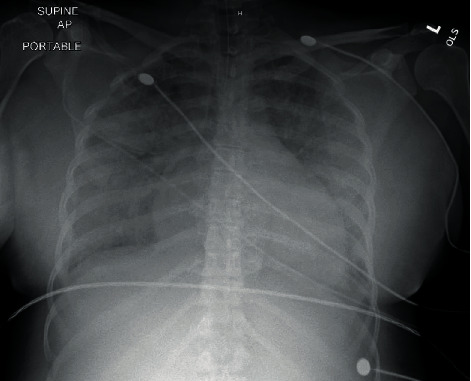
Chest X-ray demonstrating mild cardiomegaly with moderate pulmonary edema after cardiopulmonary arrest.

Later at 22:40, the patient began complaining of labored breathing while on 4 L of nasal cannula but maintained adequate oxygen saturations between 94 and 100%. Magnesium sulfate was discontinued. However, by 23:00, the patient rapidly desaturated to 82-87% on a nonrebreather, was sitting in the tripod position, and had bilateral crackles on lung auscultation. Her oxygen saturations continued to drop to 60-70%, the patient became unresponsive, and a code blue was initiated. The patient was successfully intubated, and return of spontaneous circulation was achieved after 6 minutes. She was transferred to the MICU for further care and monitoring. An A/P chest X-ray demonstrated mild cardiomegaly with moderate pulmonary edema (see Figure 1). She was then diuresed with three doses of furosemide 20 mg.

The patient's basic metabolic panel revealed a serum sodium level of 125 mMol/L, potassium level of 4.1 mMol/L, calcium level of 6.1 mg/dL, and Hgb of 8.9 mg/dL. The patient was continued on a labetalol drip to maintain blood pressures below 160/110 mmHg and was continued on furosemide 40 mg BID.

The patient improved and was successfully extubated on day 4, and her hyponatremia resolved on day 5. The net fluid of intake and output was recorded as -264 mL and -6307 mL on day 4 and day 5, respectively. She was discharged in stable condition on hydralazine 100 mg TID, labetalol 400 mg TID, hydrochlorothiazide 25 mg QD, and nifedipine 60 mg QD.

## 3. Discussion

Although the exact mechanism by which preeclampsia can induce hyponatremia is still not well understood, there are some postulated theories. Hyponatremia can be broadly categorized based on the patient's clinical volume status: hypovolemia, euvolemia, and hypervolemia [2]. Two common etiologies that have previously been described include hypervolemic hyponatremia and syndrome of inappropriate antidiuretic hormone syndrome (SIADH), leading to euvolemic hyponatremia.

Hypervolemic hyponatremia often occurs in the setting of preeclampsia, which causes a decrease in effective circulatory volume and leads to the nonosmotic release of antidiuretic hormone [3]. This mechanism mirrors conditions such as heart failure, cirrhosis, nephrosis, or hypoalbuminemia [1]. Similarly, iatrogenic causes from inappropriate fluid management and resuscitation efforts can also contribute to hypervolemic hyponatremia [1].

Preeclampsia is defined by the presence of proteinuria, which can lead to diminished perfusion with vascular hemoconcentration and a shift of fluids into the extravascular space. Furthermore, preeclampsia leads to an exaggerated inflammatory response, inappropriate endothelial activation, and vasospasms that cause increased vascular permeability. Proteinuria in preeclampsia and low colloid oncotic pressure lead to further shifting of fluids into the extravascular space. Clinically, this could lead to pulmonary edema, cerebral edema, and loss of consciousness, which occurred in this patient.

Delivery with blood loss in a preeclamptic patient can exaggerate hypoosmolality and hypovolemia secondary to the loss of plasma protein. It is important to closely monitor intake and output in the postpartum period. Excess fluid can lead to hypervolemic hyponatremia, subsequently shifting into the extravascular space, causing pulmonary edema and possible cerebral edema in preeclampsia.

Placental dysfunction has also been proposed as a mechanism of increasing ADH secretion [4]. During normal pregnancy, the placental vasopressinase enzyme acts to inactivate ADH [5]. However, during conditions such as preeclampsia leading to placental dysfunction, the levels of placental vasopressinase are decreased, leading to an inappropriate circulation of ADH [6].

Another mechanism can be explained by iatrogenic oxytocin administration, which concurrently acts on the ADH receptor due to oxytocin's molecular similarity to ADH. Given that this patient received 30 U of continuous oxytocin in 1000 mL normal saline infusion at 125 mL/h postpartum, an oxytocin-induced hyponatremia cannot be ruled out. Although elevated ADH causes euvolemic hyponatremia [7], oxytocin can still have contributory effects on patients who have developed hypervolemic hyponatremia.

Based on the clinical findings, we postulate that the main mechanism behind this patient's cardiopulmonary arrest is mainly due to hypervolemic hyponatremia secondary to postpartum fluid overload. In the setting of preeclampsia, with pulmonary capillary leakage in severe preeclampsia and low colloid oncotic pressure, hypervolemic hyponatremia can develop flash pulmonary edema and lead to noncardiogenic heart failure and cardiopulmonary arrest. The patient developed flash pulmonary edema with significant fluid overload demonstrated by her positive net intake and output values. Clinically, she appeared hypervolemic on exam with evidence of pulmonary edema on chest X-ray, which improved significantly after fluid diuresis.

Women with preeclampsia are at increased risk for developing hyponatremia; however, it is often overlooked clinically. We suggest serum sodium level should be added in routine preeclamptic laboratory tests, especially in the setting of severe preeclampsia. This particular case highlights the importance of close monitoring and early detection of hyponatremia as a preventable cause of cardiopulmonary arrest in patients with preeclampsia. The measurement of serum sodium levels is easily obtainable, cost-effective, and quickly manageable in these settings and should be emphasized within routine peripartum management in patients with preeclampsia.

## Figures and Tables

**Table 1:** Net intake and output during hospitalization.

Day	Net intake and output (mL)
1	+516
2	+5981
4	-264
5	-6307

## Data Availability

The data used to support the findings of this study are available upon request.
